# The Effects of Flavomycin and Colistin Sulfate Pre-Treatment on Ileal Bacterial Community Composition, the Response to *Salmonella typhimurium* and Host Gene Expression in Broiler Chickens

**DOI:** 10.3390/microorganisms7110574

**Published:** 2019-11-18

**Authors:** Yang He, Yanyan Yang, Yuanyang Dong, Changliang Yan, Bingkun Zhang

**Affiliations:** 1 State Key Laboratory of Animal Nutrition, Department of Animal Nutrition & Feed Science, College of Animal Science & Technology, China Agricultural University, Haidian District, Beijing 100193, China; heycau@163.com (Y.H.); yangyy6900@163.com (Y.Y.); yuanyangdong@cau.edu.cn (Y.D.); 2 Department of Basic Veterinary Science, College of Veterinary Medicine, China Agricultural University, Haidian District, Beijing 100193, China; 3 China Animal Husbandry Group, Fengtai District, Beijing 100070, China; yancl@cahg.com.cn

**Keywords:** broiler chicken, *Salmonella Typhimurium*, gene expression, intestinal bacteria, intestinal morphology

## Abstract

The composition of the bacterial community affects the intestinal health and growth performance of broiler chickens. The main purpose of this study was to explore the effects of flavomycin and colistin sulfate on the resistance to *Salmonella typhimurium* infection, ileal bacteria and intestinal health. In total, 396 1-day-old broiler chickens were randomly divided into six groups. Two groups were fed each one of the diets—the control diet (CON), the flavomycin at 10 mg/kg diet (AntiG+), and the colistin sulfate at 40 mg/kg diet (AntiG−), for 5 days. Then, one of each of the two groups was challenged with *S. typhimurium* on the 8th day; these were named CONS, AntiG+S and AntiG−S, respectively. The results showed that *S. typhimurium* significantly reduced the feed intake and body weight gain, and increased the feed conversion ratio (*p* < 0.05). It also increased the inflammatory expressions of *NF-κB* and *MyD88* genes (*p* < 0.05); and reduced the expressions of *claudin-1*, *occludin* and *mucin-2* (*p* < 0.05) tight junction genes in the intestines. *S.* *typhimurium* significantly reduced ileal bacterial diversity indexes of observed-species, chao1 and Shannon (*p* < 0.05). Compared with AntiG+S group, AntiG−S group increased the body weight gain of broiler chickens (*p* < 0.05), reduced the expression of inflammatory genes (*p* < 0.05) and intestinal permeability to fluorescein isothiocyanate (*p* < 0.05). AntiG-S group also improved the ileal bacterial diversity indexes of observed-species and Shannon (*p* < 0.05). There were many significant correlations between intestinal bacteria, intestinal gene expressions and intestinal morphology (*p* < 0.05). This study indicated that pre-constructed AntiG− bacteria could against a *S. typhimurium* infection by inhibiting the expressions of intestinal inflammation genes and increasing the diversity of intestinal bacteria.

## 1. Introduction

*Salmonella*, as a Gram-negative bacteria is a serious cause of foodborne diseases in humans and livestock around the world; it provides a significant inducement towards morbidity, mortality and economic loss in broiler production [1]. In more than 2500 *Salmonella* serotypes, *Salmonella enterica* subspecies enterica contains no less than 1500 serotypes, which includes *Salmonella typhimurium* and *Salmonella enteritidis*. This subspecies is present in more than 99% of human salmonellosis [2]. *S. typhimurium* is the most common serotype associated with livestock diseases [3,4]. The *Salmonella* infection in broiler chickens mainly causes intestinal health problems that seriously reduce growth performance and increases the spread of drug-resistant genes [5]. The increase of beneficial microorganisms in the intestines, such as *Lactobacillus* and *Bifidobacteria,* can against the *Salmonella* infections [6]. Therefore, changing the composition of gut microbes can be used as a strategy to resist the challenge of *Salmonella* and improve the growth performance of animals [7].

Both Gram-positive and Gram-negative bacteria have effects on intestinal tissue immunity and play an important role in maintaining intestinal mucous integrity, although the properties of Gram staining for bacteria are not associated with their pathogenicity. Gram staining is an important classification standard for bacteria and relates to antibiotic action. Flavomycin as a phosphorylated polysaccharide antibiotic, and its antibacterial mechanism is to inhibit the reproduction of bacteria by interfering with the biosynthesis of the structural polysaccharide peptide in the cell wall [8]. The growth-promoting principle of flavomycin may be that it improves the digestion of energy and protein in the feed [9]. The antibacterial spectrum of flavomycin is narrow, being mainly effective on Gram-positive bacteria. Its effect on Gram-negative bacteria is very weak [10]. Colistin sulfate as an alkaline peptide antibiotic, mainly used to prevent the infection of sensitive bacteria and to promote the growth of livestock and poultry [11]. Colistin sulfate can combine with the free phosphate of lipoprotein in the cell membrane. That reduces the cell membrane surface tension and increases the permeability of the membrane, resulting in cytoplasm outflow and cell death [12]. Colistin sulfate has a strong inhibitory effect on Gram-negative bacteria, especially *Escherichia* coli and *Salmonella*, while it has no effect on Gram-positive bacteria and fungi [13]. Therefore, consumption of flavomycin or colistin sulfate can specifically inhibit the growth of Gram-positive or Gram-negative bacteria to construct different intestinal bacteria.

Changing the composition of intestinal bacteria has a powerful influence on the intestinal health and the growth performance of broiler chicken. These microorganisms interact with intestinal epithelial cells to change the intestinal morphology. Studies have shown that *Salmonella* infection in broiler chicken caused intestinal immune responses and improved the inflammation-related gene expressions of *IFNγ*, *IL-12*, and *IL-18* [14]. The resistance to *Salmonella* can be improved by regulating the intestinal bacteria [6]. A large number of previous studies have focused on the therapeutic effect of antibiotics against *S. typhimurium* infection [15,16]. There has been no such research on the resistance of *S. typhimurium* through the early construction of different intestinal bacteria.

Therefore, the purpose of this study was to explore the effects of Gram-negative or positive intestinal bacterial communities constructed by flavomycin or colistin sulfate, on the growth performance, intestinal bacteria, intestinal morphology and expression of intestinal mucosal genes in broiler chickens. At the same time, the resistances of these constructed, different intestinal bacterial communities to *Salmonella* infection were studied.

## 2. Materials and Methods

### 2.1. Experimental Design and Sample Collection

The animal protocol was approved by Chinese Agricultural University Laboratory Animal Welfare and Animal Experimental Ethical Committee (permit number AW16109102-1, 15 September, 2014). In this experiment, a total of 396 1-day-old Arbor Acres broiler chickens were divided into six treatment groups, each with 6 repetitions of 11 broiler chickens. The three diets used consisted of a control diet (Table 1), flavomycin at 10 mg/kg in the control diet, and colistin sulfate at 40 mg/kg in the control diet. For each diet, two groups of broiler chickens were assigned, for 5 days. Then, the treatment diets were replaced with the control diet to build the intestinal bacteria of—the control group (CON), anti-gram-positive bacteria group (Anti G+) and anti-Gram-negative bacteria group (Anti G−). On the 8th day of feeding, one of each of the above two groups for the three diets were administered 1 × 10^9^ CFU of *S. typhimurium* per chicken, and named CONS, Anti G+S and Anti G−S, respectively. The temperature in the bird room was kept at about 35 °C for the first week; then, it gradually dropped and remained at 25 °C. The relative humidity of the environment was maintained at 65–70%. The light remained for 24 h during the first 3 days; that was followed by 23 h of light and 1 h of darkness. The number of dead chickens was recorded every day. At the 14th, 21st and 42th days, the feed intake, body weight gain, feed conversion ratio and mortality rate were calculated.

On the 14th and 21st days, one chicken was randomly selected to be weighed and slaughtered from each repetition. The ileal content was collected and frozen by liquid nitrogen and stored in −80 °C for 16S rDNA Sequencing. About a 0.5 cm terminal ileum segment was cut and fixed in 4% polyformaldehyde solution in each case. The mucosal tissues of the intestinal segments were gently scraped with glass slides. The mucosal tissues were frozen with liquid nitrogen and stored in −80 °C for Real-time PCR.

### 2.2. DNA Extraction and 16S rDNA Sequencing of Ileal Microbes

The DNA of ileal contents on the 21st day was extracted by TIANamp Stool DNA Kit (Tiangen Biotech, Beijing, China). The DNA concentration was determined by Nano Drop 2000 (Thermo Fisher Scientific Inc., DE, USA). Two samples in the same treatment group were mixed with an equivalent amount of DNA to obtain a representative sample, so that each group had three representative samples for sequencing. The V3–V4 hypervariable region of the 16S rDNA was sequenced and analyzed by Beijing Nohe Bioinformatics Technology Co., Ltd. The sequencing data was analyzed according to QIIME processes [17].

The raw sequencing data were submitted to the Sequence Read Archive (SRA) of NCBI, and the SRA accession number is PRJNA577381.

### 2.3. Ileum Histomorphology

The paraffin embedded tissues were cut into thicknesses of 0.5 μm. Each glass slide reflects the morphology of an about 0.5 cm intestinal segment. The slides were processed and stained with hematoxylin-eosin (H&E). The sections were observed and analyzed by SmartV350D analysis system (Jieda Technology Development Co Ltd., Jiangsu, China). Ten intact intestinal villi were randomly selected for each section. The height of each intestinal villus and the corresponding crypt depth were determined. The villus height was based on the vertical height from the opening of the intestinal gland to the top of the villus. The crypt depth was based on the vertical height from the muscular layer of the mucosa to the opening of the intestinal gland.

### 2.4. Detection of Ileal Permeability by Serum Method

On the 14th day, one chicken was randomly selected from each repetition. The intestinal segment, about 2 cm before the junction of the ileum and cecum, was taken and placed in a silica gel dish which contained Hank’s balanced salt solution (HBSS) buffer. The gas composition 95% O_2_ and 5% CO_2_ was maintained. The intestinal segment was cut and rinsed to remove the intestinal contents. Then, the intestinal serosa layer was stripped to keep the mucosa intact. The intestinal segments were loaded into the Ussing Chamber splint and placed in the compartment. The mucosal side compartment was injected with 3.5 mL HBSS buffer containing 2 mg/mL fluorescein isothiocyanate-dextran (FITC), and the serosal side was injected with the same volume of HBSS buffer. 300 μL solution in serosal side was taken after 60 min. The ileal permeability was measured by a fluorescent-concentrate of FITC-dextran under a 485/525 nm dual-wavelength multifunctional microplate reader. On the 21st day, one chicken was randomly selected from each group. The chickens were injectedwith 1 mL of 10 mg/mL FITC-dextran solution. The blank control group was injected with the same volume of distilled water. After 3 h, venous sinus blood was collected to separate the serum by 3000× *g* centrifuging for 10 min. The FITC-dextran fluorescence-concentration standard curve was calculated under 485/525 nm dual-wavelength multifunctional microplate reader, and the FITC-dextran content in serum was detected. Intestinal permeability was tested according to previous report [18].

### 2.5. Myeloperoxidase (MPO) Activity in the Ileal Mucosa

On the 14th and 21st days, one chicken was randomly selected from each repetition. The middle and posterior segments of the ileum were cut to open by scissors. The mucosal tissues of the intestinal segments were gently scraped with glass slides and weighed in centrifugal tubes. The chickens were frozen with liquid nitrogen and then transferred to a freezer at −80 °C for preservation. The MPO activity was determined in accordance with the corresponding kit (Nanjing Jiancheng Biological Company, Nanjing, China).

### 2.6. Total RNA Extraction and Quantitative Real Time PCR

The RNA of ileal tissue was extracted by Trizol according to the instructions (Invitrogen Life Technologies, Carlsbad, CA, USA). Each group had six repetitions. The concentration and quality of RNA were measured by Nano-300 spectrophotometer (Yuanpinghao Biotechnology Co., Ltd., beijing, China). The reverse transcription of RNA was performed by Revert Aid^TM^ First Strand Gene Synthesis Kit (Takara Biotechnology Co., Ltd., Dalian, China). The real-time quantitative PCR was performed by ABI7500 real-time quantitative PCR instrument (Applied Biosystems Inc, Foster City, CA, USA) and SYBR Premix EX TaqTM II (Takara Biotechnology Co., Ltd., Dalian, China). *GAPDH* was used as internal reference gene. The relative expression of other genes were calculated according to the former research [19]. The primer sequences used to determine the expressions of *claudin-1*, *occludin*, *mucin-2*, *IFN-γ*, *TLR2*, *TLR4*, *NF-κB*, *MyD88* and *GAPDH* were designed by Primer-BLAST (https://www.ncbi.nlm.nih.gov/tools/primer-blast) of NCBI and are listed in the Table 2.

### 2.7. Statistical Analysis

The data were analyzed by two-way ANOVA using the GLM in SPSS17.0 software (IBM Corporation, Armonk, NY, United States). The difference between the main effect and the interaction effect was tested; *p* < 0.05 indicated that the difference was significant. Duncan’s test was used to make multiple comparisons among groups in the presence of interaction effects. The spearman rank correlation analysis of the relative abundance of intestinal bacteria and other indicators of the intestines was carried out by the packages Corrplot and Gplots in R for Windows 3.5.1 software (The R Foundation for Statistical Computing, Vienna, Austria).

## 3. Results

### 3.1. Growth Performance

For days 1–14, the feed intake and body weight gain in the Anti G− group were significantly higher than that in the Anti G+ group. *S. typhimurium* increased the feed conversion ratio (Table 3). *S. typhimurium* reduced body weight gain and feed intake for days 1–21 (*p* < 0.05). The intestinal bacterial community and *S. typhimurium* had an interaction effect on the feed conversion ratio (*p* < 0.05). The CONS group was significantly higher than the Anti G− group (*p* < 0.05). The feed conversion ratio of the diet and *Salmonella* in days 1 to 42 had a significant interaction effect. The feed conversion ratio of the CON group was significantly lower than that of other groups (*p* < 0.05), but the difference between CON and Anti G−S was not significant.

### 3.2. Ileal Morphology

The effects of the intestinal bacterial community on the intestinal morphology are shown in Table 4. The differences of the villus height and the crypt depth among the treatment groups were not significant. The ratio of villus height to the crypt depth (V/C) in the CON group was significantly higher than that in the Anti G− group on the 14th day (*p* < 0.05). *S. typhimurium* significantly reduced the V/C measurements on the 14th day and 21st day (*p* < 0.05).

### 3.3. Ileal Gene Expression on the 14th Day

On the 14th day, the effects of intestinal bacterial communities and *S. typhimurium* on gene expressions in the intestines are shown in Table 5. The intestinal bacterial community can significantly affect the expressions of *mucin-2*, *IFN-γ* and *NF-κB*. The Anti G+ group had the highest expression of *mucin-2* gene, while the CON group had the highest expression of the *IFN-γ* gene. The Anti G− group had the highest expression of the *NF-κB* gene. *S. typhimurium* significantly inhibited the gene expressions of *claudin-1*, *occludin*, *NF-κB* and *MyD88* (*p* < 0.05), and significantly increased the gene expressions of *IFN-γ*(*p* < 0.05).

### 3.4. Ileal Gene Expression on the 21st Day

On the 21st day, the type of intestinal bacterial community and the presence of *S. typhimurium* had an interactive effect on *mucin-2*, *IFN-γ* and *TLR2* gene expressions (Table 6). The expression of the *mucin-2* gene in the Anti G−S group was significantly higher than that in the other treatment group, except in the Anti G−S group (*p* < 0.05). The *IFN-γ* gene expression in Anti G− group was significantly higher than in the CONS and Anti G−S groups (*p* < 0.05). The expression of the *TLR2* gene in the Anti G−S group was significantly higher than that in Anti G+ and Anti G− groups (*p* < 0.05). *S. typhimurium* significantly inhibited the gene expressions of the *claudin-1* and *occludin*, and increased the *TLR4* gene’s expression (*p* < 0.05).

### 3.5. Intestinal Permeability and MPO Activity

**Figure 1:**
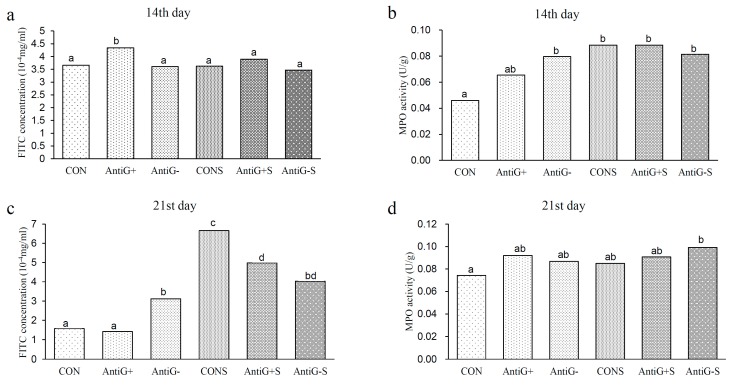
The effects of intestinal bacterial communities on the mucosal permeability of fluorescein isothiocyanate (FITC) on the 14th (**a**) and 21st days (**c**), and myeloperoxidase (MPO) activity on the 14th (**b**) and 21st (**d**) days in broiler chickens. Small alphabetic letters show significance (*p* < 0.05).

The results of intestinal permeability and MPO activity are presented in Figure 1. On the 14th day, the intestinal permeability in the Anti G+ group was significantly higher than in the other groups (*p* < 0.05). The MPO activity in the CON group was significantly lower than in the other groups, except for the Anti G+ group (*p* < 0.05). On the 21st day, the difference in intestinal permeability between the CON and the Anti G+ groups was not significant, while the intestinal permeabilities in the Anti G− group and the *S. typhimurium* group were significantly higher than those in the CON and Anti G+ groups, and the MPO activity in the CON group was significantly lower than that in the Anti G−S group (*p* < 0.05).

### 3.6. Alpha Diversity Analysis

**Figure 2:**
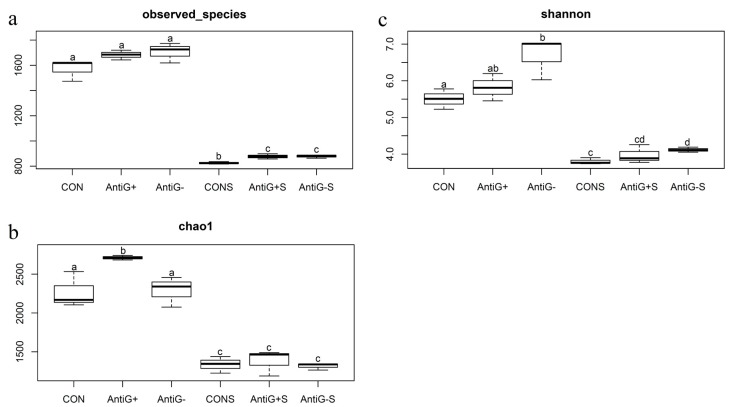
The effects of intestinal bacterial community on the ileal bacterial community diversity indexes of observed_species (**a**), chao1 (**b**), and Shannon (**c**) on the 21st day. Small alphabetic letters show significance when (*p* < 0.05).

The effect of diet on the ileal bacterial diversity index is shown in Figure 2; the three diversity indices in *S. typhimurium* groups were significantly lower when compared to the unfilled *S. typhimurium* groups (*p* < 0.05). The observed_species index in the CONS group was significantly lower than in the Anti G−S and Anti G+S groups (*p* < 0.05). The chao1 index in Anti G+ group was significantly higher than in the CON and Anti G− groups (*p* < 0.05). The Shannon index in the Anti G− group was significantly higher than that in the CON group, while the CONS group had one significantly lower than the Anti G−S group’s (*p* < 0.05).

### 3.7. Taxonomic Analysis

**Figure 3:**
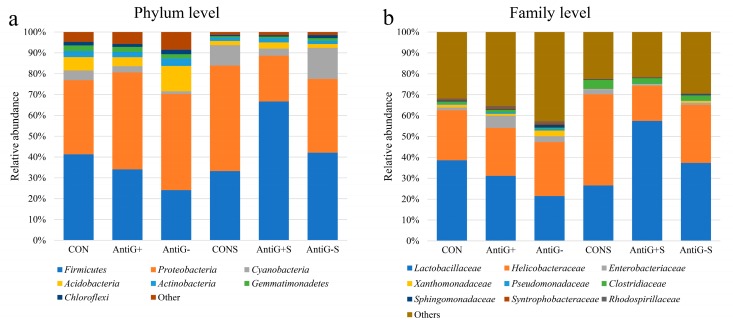
The effects of intestinal bacterial communities on the relative abundances of ileal bacteria at the phylum level (**a**) and family level (**b**) on the 21st day.

The relative abundance of ileal bacteria in each treated group are presented in Figure 3. *Firmicutes* and *Proteobacteria* were the predominant bacteria at the phylum level, accounting for about 40.2% and 39.4% of the total bacteria, respectively. There were significant differences in the relative abundance of the two main bacteria in each group. Compared with the CON group, the Anti G− and Anti G+ groups had lower relative abundance of *Firmicutes* and higher relative abundance of *Firmicutes*. The Anti G+S and Anti G−S groups increased the relative abundance of *Firmicutes* and reduced the relative abundance of *Proteobacteria*. The Anti G− group had the lowest abundance of *Firmicutes* which accounted for about 24%. The Anti G−S had the highest abundance of *Firmicutes* about 66% and the lowest relative abundance of *Proteobacteria* accounted for about 22%. The CONS group had the highest relative abundance of *Proteobacteria* phylum.

At the family level, *Lactobacillaceae* and *Helicobacteraceae* were the main dominant bacteria, accounting for 35.4% and 26.8% of all the samples, respectively. The Anti G− and Anti G+ groups had lower *Lactobacillaceae* family compared to the CON group. The relative abundance of *Lactobacillaceae* in Anti G−S and Anti G+S increased after the challenge with *S. typhimurium*. *Lactobacillaceae* in the Anti G− group had the lowest proportion at 21.4%, while the Anti G−S group had the highest proportion at 57.4%. *Helicobacteraceae* in the Anti G−S group had the lowest proportion at 16.7%, while the CONS group had the highest proportion at 43.6%.

### 3.8. Beta Diversity

**Figure 4:**
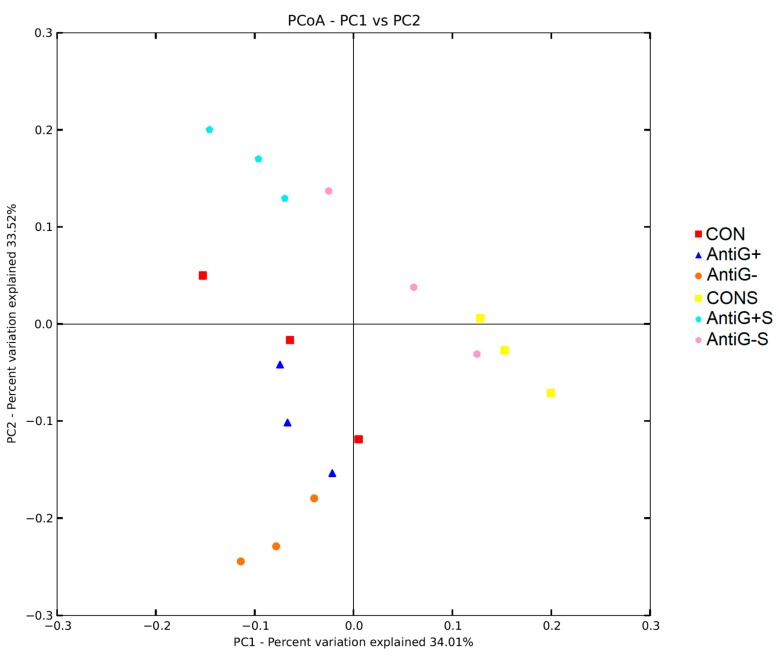
Principal component analysis (PCA) of ileal bacteria communities based on weighted UniFrac distances on the 21st day.

The principal component analysis (PCA) of the treatments showed significant clustering between unfilled *S. typhimurium* groups and *S. typhimurium* groups. PC1 and PC2 showed 34.01% and 33.52% of the explanatory degrees, respectively (Figure 4). At the same time, the PCA also showed clustering of the bacteria in each diet treatment group.

### 3.9. Correlation Analysis

**Figure 5:**
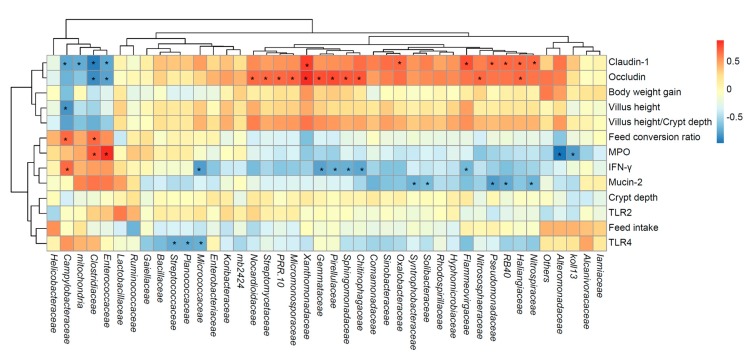
The correlations between the ileal bacteria communities and intestinal genes’ expressions. The lattices were colored based on Spearman’s rank correlation analysis. A red cell indicates a positive correlation, and the blue color cell indicates a negative correlation. * indicates a significant correlation (*p* < 0.05).

The correlation analysis between the relative abundances of intestinal bacteria and the expressions of intestinal genes is shown in Figure 5. Intestinal genes’ expressions were associated with the relative abundances of a variety of gut bacteria. *Occludin*, *claudin-1*, *mucin-2* and *IFN-γ* were significantly related to the multiple bacteria in the ilea, while the feed intake, body weight gain and crypt depth were not significantly related to the gut bacteria.

## 4. Discussion

There is no doubt that the chicken model has been used for the studies of different diseases. But little was known about the resistance to *S. typhimurium* infections in different intestinal bacterial structures of broiler chickens during the early stages. Prior to *S. typhimurium* infection in this study, flavomycin and colistin sulfate were used to create intestinal Anti G+ and Anti G− chickens, respectively.

Although some studies have shown the positive effects of antibiotic growth promoters on poultry production [15,20], this study found that short-term supplementation of antibiotic growth promoters in the early stages did not improve growth performance. That has also been reported before [21]. However, compared with the Anti G+ group, the Anti G− group can increase body weight gain and feed intake. This may be due to the ability of the Anti G− group to suppress the infection of *S. typhimurium*, which belongs to the Gram-negative group. The results of the present study substantiated the adverse impacts of *S. typhimurium* on the growth performance of the broiler chickens; parameters indicating that included body weight gain, feed intake and feed conversion ratio. Our values were similar to those of previous findings [14,22]. *S. typhimurium* infection in several day old chicks can cause a severe inflammatory response and intestinal lesions [23]. However, since the immune system improve and the gut microbes mature, older chickens can resist *S. typhimurium* infection. Therefore, *S. typhimurium* can have different results in different gut microbiome structures. In the results of this study, compared with the Anti G+ group, the intestinal bacterial community of Anti G− was more conducive to protecting gut health against *S. typhimurium* infection and improving the growth performance.

In this study, the Anti G+ and Anti G− bacterial communities were not able to promote the improvement of intestinal villus height and crypt depth, which departs from previous studies’ results. Other studies have shown that the whole long-term feeding of flavomycin can increase villus height [15] or have no effect [24]. The Anti G+ group built in the first 5 days did not change the intestinal shape. Although the Anti G− reduced the ratio of villus height to crypt depth by the 14th day, the difference was not significant on the 21st day. This shows that the pre-constructed intestinal bacterial community has a negative effect on intestinal morphology, but the effect of the pre-stage was weakening with increasing age. The *S. typhimurium* reduced the ratio of villus height to crypt depth, which indicated that *S. typhimurium* infection inhibited the digestion and absorption of nutrients, leading to a reduction in feed conversion ratio.

The Toll-like receptors (TLRs) 2 and 4 are the receptors for lipopolysaccharides, which are produced by Gram-negative bacteria in the cytoderm, such as *S. typhimurium*. Those lead to a surplus in the production of pro-inflammatory cytokines by starting and activating the downstream signaling cascades, such as nuclear factor-κB (*NF-κB*) [25,26]. *MyD88* can be used as an adapter to transmit upstream and downstream connections during the above process [27]. In this study, both the intestinal bacterial community and the infection of *S. typhimurium* had effects on the *NF-κB* gene’s expression and performed differently between 0–14 and 14–21 days. Compared to the control group, Anti G− promoted *NF-κB* gene expression on the 14th day, while Anti G+ increased this gene expression on the 21st day. This shown that both Anti G- and Anti G+ can cause inflammation of the intestines through the repression of *TLR4/MyD88/NF-κB* signaling pathway. Gene *IFN-γ* is important in the immune response of the intestinal epithelial, and *S. typhimurium* induces these gene expressions. Although inflammation is protective to *S. typhimurium* infection, uncontrolled inflammatory response can lead to tissue damage and high nutritional consumption [28]. The result was in accordance with recent studies [14,29]. The Anti G− bacteria can promote intestinal health by inhibiting the inflammatory response of the intestines.

The integrity of intestinal mucosa and the perfection of intestinal epithelial cell function are of great significance to maintain intestinal health and ensure animal production performance. *Mucin-2* as the main component of the intestinal mucus, is produced mainly by goblet cells in small and large intestinal epithelial cells. It plays a key protective function [30]. The *claudin-1* and *occludin* as the mucous membrane epithelial tight junctions has an important role to prevent microorganisms and toxins from invading animal intestinal tissue [31]. The Anti G− and Anti G+ constructed in the early part of this study had no effect on the damage of the tight junctions of *claudin-1* and *occludin*. *S. typhimurium* infection were in accordance with previous study [32], the Anti G+ promoted the expression of mucoprotein genes during the *S. typhimurium* infection.

MPO, as a derivative leucocyte enzyme, can catalyze the production of many reactive oxidants and diffusible radical substances [33]. MPO efficiently removes the spread H_2_O_2_ in the surface of phagosomal *S. typhimurium* and converts it into a highly active HOCl [34]. In the current study, the immune response caused by *S. typhimurium* increased the MPO level. The tight junction damage of intestinal epithelial cells, which is caused by the inflammatory reaction to *S. typhimurium* infection, can led to an increase in the permeability of epithelial cells. That was evidenced by the increase of FITC concentration.

Diets play a key role in regulating intestinal bacteria, the substances that are not digested and absorbed by the host can provide nutrients to gut bacteria. At the same time, complex and perfect gut microbes are essential for the intestinal immunity and health of broiler chickens. The diversity of intestinal bacteria is beneficial to maintaining the balance of intestinal bacteria, to maintaining the stability of the environment in the intestines and to resisting the invasion of pathogenic bacteria. *S. typhimurium* infection in this study significantly reduced the diversity index of enterobacteria, suggesting that *S. typhimurium* not only caused inflammation of the gut itself, but also improved the intestinal susceptibility by reducing the diversity of gut bacteria. It is worth noting that the observed_species and Shannon diversity indices in Anti G−S were significantly higher than those in the CONS group, indicating that Anti G− bacteria were able to defend against *S. typhimurium* infection to some extent by maintaining the balance of intestinal bacteria.

On the composition of ileal bacteria, the data in this study are consistent with the results of most previous studies [35,36]; the Firmicutes and Proteobacteria phyla are the main advantageous ones in broiler chicken [37,38]. Unlike in previous studies, the relative abundance of Bacteroidetes in this study was less than 1%. Moreover, Firmicutes, being the main Gram-positive bacteria in the Anti G+ group, was not the least present. Proteobacteria, as the main Gram-negative bacteria in the Anti G− group proportion was not the least present. Those results indicated that the pre-constructed bacterial community was not always invariant, especially during *S. typhimurium* infection. Cyanobacteria, as the third largest bacterial phylum in the *S*. typhimurium group, was significantly higher than that in the unfilled *S. typhimurium* group; the relative abundance of those bacteria was reduced under heat stress [39].

At the family level, *Lactobacillaceae* was the dominant family in the ileum of the broiler chicken, which has previously been reported [40]. *Lactobacillaceae* can biosynthetic biotin to inhibit the proliferation of other bacteria. Moreover, it can ferment to produce lactic acid, reduce intestinal pH, inhibit the activity of pathogenic bacteria such as *E. coli* [41,42]. In this study, Anti G− and Anti G+ can reduce the relative abundance of *Lactobacillaceae*. While the Anti G−S and Anti G+S groups can increase the relative abundance of *Lactobacillaceae*. This may be one of the reasons that the *S. typhimurium* group had a lower diversity index, because *Lactobacillaceae* reduces the number of other bacteria. The difference in the relative abundance of these dominant bacteria directly led to a significant classification between *S. typhimurium* groups and unfilled *S. typhimurium* groups in PCA.

In the correlation analysis between the intestinal bacterial community and the intestinal gene expression. It is interesting that the three main dominant bacterial families were not significantly related to any indicator, indicating that the intestinal health is not regulated by a single dominant bacterium. The bacteria of high abundance have a more complex effect on the intestine. Those significant correlation may be due to the direct or indirect involvement of microorganisms on pathways that affect gene expression and intestinal cell metabolism. For example, there was a positive correlation between *Xanthomonadaceae* family and gene expression of *Claudin-1* and *Occludin*. Studies have shown that the members of *Xanthomonadaceae* family are the main bacteria in the intestinal mucosa and can promote the activity of esterase, lipase, and urease in the hindgut [43,44]. These effects can improve the expression of closely connected genes.

## 5. Conclusions

The results of this study show that the Anti G+ and Anti G− intestinal bacterial communities, when constructed in the early stages, can react differently to *S typhimurium* infection. *S. typhimurium* infection in broiler chicken can reduce the diversity index of intestinal bacteria, cause a wide range of inflammatory reactions and reduce the production performance. The above-mentioned adverse symptoms can be effectively alleviated by building the bacterial community in the early stage. An Anti G− intestinal bacterial community can increase the diversity of an intestinal bacterial community to improve the stability of intestinal bacteria. Anti G− also inhibited the inflammatory response that is caused by *S. typhimurium*. Anti G−S improved the tight junctions of the intestinal epithelia, reduced the permeability of FITC and ultimately increased body weight gain.

## Figures and Tables

**Table 1:** The nutrient composition of the control diet.

Ingredients (%)	Content	Chemical Composition	Content
Yellow corn	56.34	ME (Kcal/kg)	2.96
Soybean meal	33.5	Crude protein (%)	21
Corn gluten meal (60%)	3	Calcium (%)	1
Soybean oil	2.9	Nonphytate P (%)	0.45
Limestone	1.25	Tryptophan	0.23
Dicalcium phosphate	1.9	Methionine (%)	0.5
Vitamin premix ^1^	0.02	Lysine (%)	1.15
Mineral premix ^2^	0.2	Threonine (%)	0.78
Salt	0.35		
dl-methionine	0.19		
l-lysine Hcl	0.12		
Choline chloride	0.2		
Ethoxyquin	0.03		

^1^ The trace mineral premix provides the following per kg in the diet: copper: 8 mg; ferrum: 80 mg; manganese: 100 mg; selenium: 0.15 mg; iodine: 0.35 mg. ^2^ The vitamine premix provides the following per kg in the diet: vitamin A: 9500 IU; vitamin D3: 62.5 µg; vitamin E: 30 IU; vitamin K_3_: 2.65 mg; vitamin B_1_: 2 mg; vitamin B_6_ 6 mg; vitamin B12: 0.025 mg; biotin: 0.0325 mg; folic acid: 1.25 mg; pantothenic acid: 12 mg; nicotinic acid: 50 mg.

**Table 2:** The primer sequences used for quantitative real time PCR.

Gene Name	Primer Sequence (5‘–3′)	GenBank
*claudin-1*	F: CATACTCCTGGGTCTGGTTGGT	AY750897.1
	R:GACAGCCATCCGCATCTTCT	
*occludin*	F:ACGGCAGCACCTACCTCAA	D21837.1
	R:GGGCGAAGAAGCAGATGAG	
*mucin-2*	F:TTCATGATGCCTGCTCTTGTG	XM_421035
	R:CCTGAGCCTTGGTACATTCTTGT	
*IFN-γ*	F:AGCTGACGGTGGACCTATTATT	Y07922
	R:GGCTTTGCGCTGGATTC	
*TLR2*	F:CTGGGAAGTGGATTGTGGA	AB050005.2
	R:AAGGCGAAAGTGCGAGAAA	
*TLR4*	F:AGTCTGAAATTGCTGAGCTCAAAT	NM-001030693
	R:GCGACGTTAAGCCATGGAAG	
*NF-κB*	F:GTGTGAAGAAACGGGAACTG	NM-205129
	R:GGCACGGTTGTCATAGATGG	
*MyD88*	F:CTGGCATCTTCTGAGTAGT	NM-001030962
	R:TTCCTTATAGTTCTGGCTTCT	
*GAPDH*	F:CCTAGGATACACAGAGGACCAGGTT	NM-204305
	R:GGTGGAGGAATGGCTGTCA	

**Table 3:** The effects of intestinal microflora on growth performance in broiler chickens.

Treatment		1–14 d			8–14 d	1–21 d			15–21 d	1–42 d			1–42 d
		Body Weight Gain (g)	Feed Intake (g)	Feed Conversion Ratio	Mortality (%)	Body Weight Gain(g)	Feed Intake(g)	Feed Conversion Ratio	Mortality (%)	Body Weight Gain (g)	Feed Intake (g)	Feed Conversion Ratio	Mortality (%)
CON		242.98	333.47	1.39	0	533.26	879.29	1.63 ^ab^	0	2436.8	5145.4	2.09 ^a^	0
Anti G+		220.24	321.9	1.42	0	499.96	844.23	1.68 ^ab^	0	2431.8	5293.3	2.2 ^b^	0
Anti G−		244.29	346.33	1.4	0	549.74	893.06	1.59 ^a^	0	2412.5	5321.0	2.24 ^b^	0
CONS		222.3	321.77	1.47	0	483.93	808.05	1.72 ^b^	3.03	2365.9	5093.8	2.24 ^b^	3.85
Anti G+S		222.96	319.23	1.43	1.52	490.47	804.4	1.65 ^ab^	0	2324.3	5281.5	2.22 ^b^	2.56
Anti G−S		239.52	337.05	1.44	0	511.35	865.56	1.68 ^ab^	0	2386.9	5045.5	2.15 ^ab^	0
SEM		3.14	3.18	0.01	0	6.39	11.37	0.01	0	19.38	37.37	0.02	0.01
**Main effect**													
Bacterial community	CON	232.64 ^ab^	327.62 ^ab^	1.43	0	508.59 ^ab^	843.67	1.68	1.52	2401.4	5119.6	2.17	1.93
	Anti G+	221.60 ^a^	320.56 ^a^	1.43	0.76	495.22 ^a^	824.31	1.67	0	2378.0	5287.4	2.21	1.28
	Anti G−	241.90 ^b^	341.69 ^b^	1.42	0	530.55 ^b^	879.31	1.64	0	2399.7	5183.2	2.20	0
*S. typhimurium*	−	235.32	334.76	1.4	0	527.25	873.8	1.64	0	2426.9	5259.5	2.18	0
	+	227.89	326.7	1.45	0.01	495.59	822.17	1.69	0.01	2361.5	5155.9	2.20	2.14
***p* value**													
Feed		0.02	0.019	0.902	0.38	0.027	0.114	0.253	0.099	0.895	0.166	0.504	0.436
*S. typhimurium*		0.182	0.184	0.005	0.325	0.004	0.035	0.012	0.124	0.114	0.156	0.435	0.091
Bacterial community * *S. typhimurium*		0.233	0.815	0.135	0.38	0.281	0.668	0.02	0.099	0.711	0.295	0.007	0.436

Means in the same row with different superscript letters differ significantly (*p* < 0.05). Bacterial community * *S. typhimurium*: the interaction effect between the bacterial community and *S. typhimurium.*

**Table 4:** The effects of intestinal bacterial communities on ileal morphology in broiler chickens.

Treatment		14 d			21 d		
		Villus Height (μm)	Crypt Depth (μm)	V/C	Villus Height (μm)	Crypt Depth (μm)	V/C
CON		561.84	155.89	3.67	600.61	166.34	3.66
Anti G+		544.6	159.13	3.51	571.76	160.8	3.64
Anti G−		557.2	159.86	3.52	571.92	160.36	3.52
CONS		554.1	164.33	3.49	551.91	157.29	3.42
Anti G+S		548.22	157.3	3.47	554.59	163.61	3.47
Anti G−S		524.91	157.15	3.44	592.39	176.23	3.42
SEM		5.16	1.61	0.02	6.37	2.32	0.03
**Main effect**							
Bacterial community	CON	557.97	157.52	3.58 ^a^	570.95	161.81	3.54
	Anti G+	550.86	158.22	3.49 ^ab^	563.18	161.04	3.54
	Anti G−	543.01	155.36	3.48 ^b^	582.15	169.5	3.48
*S. typhimurium*	−	554.14	158.08	3.57	580.69	162.13	3.61
	+	541.57	160.08	3.47	566.30	166.31	3.44
***p* value**							
Feed		0.421	0.875	0.025	0.418	0.421	0.358
*S. typhimurium*		0.253	0.702	0.002	0.219	0.482	0.003
Bacterial community * *S. typhimurium*		0.381	0.305	0.184	0.090	0.109	0.494

Means in the same row with different superscript letters differ significantly (*p* < 0.05). V/C: the ratio of villus height to the crypt depth. Bacterial community * *S. typhimurium*: the interaction effect between the bacterial community and *S. typhimurium.*

**Table 5:** The effects of intestinal bacterial communities on tight junction, *mucin-2* and immune-related genes’ expressions in broiler chickens on the 14th day.

Treatment		*claudin-1*	*occludin*	*mucin-2*	*IFN-γ*	*TLR2*	*TLR4*	*NF-κB*	*MyD88*
CON		1.03	1.02	1.02 ^ab^	1.02	1.04	1.02	0.9	1.01
Anti G+		0.8	0.84	0.99 ^ab^	1.25	1.01	1.35	0.85	0.86
Anti G−		0.87	1.11	0.89 ^a^	1.24	0.87	1.18	1.06	0.94
CONS		0.51	0.77	1.67 ^c^	1.21	1.06	1.26	1.69	1.58
Anti G+S		0.43	0.75	2.12 ^d^	1.52	1.39	1.26	1.91	1.59
Anti G−S		0.62	0.79	1.35 ^bc^	1.28	0.98	1.24	1.95	1.71
SEM		0.05	0.03	0.09	0.04	0.05	0.04	0.10	0.07
**Main effect**									
Bacterial community	CON	0.77	0.89	1.34 ^ab^	1.11 ^a^	1.05	1.14	1.33 ^b^	1.24
	Anti G+	0.62	0.79	1.55 ^a^	1.38 ^b^	1.2	1.3	1.39 ^ab^	1.18
	Anti G−	0.74	0.95	1.12 ^b^	1.26 ^ab^	0.92	1.21	1.55 ^a^	1.32
*S. typhimurium*	−	0.91	0.98	0.97	1.15	0.98	1.17	0.95	0.94
	+	0.53	0.77	1.69	1.32	1.13	1.25	1.86	1.62
***p* value**									
Feed		0.203	0.060	<0.001	0.024	0.055	0.182	0.040	0.469
*S. typhimurium*		<0.001	0.001	<0.001	0.035	0.058	0.342	<0.001	<0.001
Bacterial community * *S. typhimurium*		0.268	0.164	0.004	0.499	0.222	0.194	0.281	0.393

Means in the same row with different superscript letters differ significantly (*p* < 0.05). Bacterial community * *S. typhimurium*: the interaction effect between the bacterial community and *S. typhimurium.*

**Table 6:** The effects of differing intestinal bacterial communities on tight junction, *mucin-2* and immune-related genes’ expressions in broiler chickens on the 21st day.

Treatment		*claudin-1*	*occludin*	*mucin-2*	*IFN-γ*	*TLR2*	*TLR4*	*NF-κB*	*MyD88*
CON		1.04	1.02	1.02 ^a^	1.04 ^a^	1.03 ^ab^	1.01	1.01	1.02
Anti G+		1.12	0.93	1.20 ^a^	1.37 ^ab^	0.88 ^a^	1.24	1.33	1.27
Anti G−		0.89	0.94	1.28 ^a^	1.26 ^a^	0.95 ^a^	1.1	1.09	1.19
CONS		0.71	0.69	1.01 ^a^	1.74 ^b^	1.08 ^ab^	1.32	0.98	0.96
Anti G+S		0.52	0.78	1.74 ^b^	1.80 ^b^	1.32 ^a^	1.54	1.15	1.27
Anti G−S		0.64	0.93	1.40 ^ab^	1.32 ^ab^	1.15 ^ab^	1.5	1.1	1.06
SEM		0.05	0.04	0.06	0.06	0.04	0.05	0.03	0.03
**Main effect**									
Bacterial community	CON	0.88	0.85	1.01 ^a^	1.39 ^ab^	1.06	1.17	1.00 ^a^	0.99 ^a^
	Anti G+	0.82	0.85	1.47 ^b^	1.59 ^a^	1.1	1.39	1.24 ^b^	1.27 ^b^
	Anti G−	0.77	0.93	1.34 ^b^	1.29 ^b^	1.05	1.3	1.11 ^ab^	1.12 ^ab^
*S. typhimurium*	−	1.02	0.96	1.16	1.22	0.95	1.11	1.14	1.15
	+	0.63	0.81	1.35	1.62	1.18	1.46	1.08	1.10
***p* value**									
Feed		0.448	0.481	<0.001	0.026	0.742	0.054	0.009	<0.001
*S. typhimurium*		<0.001	0.017	0.008	<0.001	0.001	<0.001	0.299	0.256
Bacterial community * *S. typhimurium*		0.137	0.135	0.018	0.012	0.045	0.841	0.426	0.568

Means in the same row with different superscript letters differ significantly (*p* < 0.05). Bacterial community * *S. typhimurium*: the interaction effect between the bacterial community and *S. typhimurium.*
